# A Pathogenic *Providencia rettgeri* Isolated and Identified from *Pelodiscus sinensis*

**DOI:** 10.3390/vetsci12121207

**Published:** 2025-12-16

**Authors:** Yan Meng, Mingyang Xue, Nan Jiang, Chunjie Zhang, Wei Liu, Tong Zhou, Yuding Fan, Ke Jin, Zidong Xiao, Yong Zhou

**Affiliations:** 1 Yangtze River Fisheries Research Institute, Chinese Academy of Fishery Sciences, Wuhan 430223, China; 2 College of Animal Science and Technology, Yangzhou University, Yangzhou 225000, China

**Keywords:** Chinese soft-shelled turtle (*Pelodiscus sinensis*), *Providencia rettgeri*, isolation and identification, pathogenicity

## Abstract

The infectious diseases have become more frequent with the production of farmed Chinese soft-shelled turtles (*Pelodiscus sinensis*) increasing. In this study, bacterial strain was isolated, identified, and characterized from the liver of diseased Chinese soft-shelled turtles exhibiting body surface hemorrhages, claws and tail tips necrosis, and bleeding spots in visceral tissues. Based on the analysis results of morphology, biochemistry, and 16S ribosomal RNA (16S rRNA) sequencing, one bacterium *Providencia rettgeri* (*P. rettgeri*) was identified. The histopathological examination results revealed varying degrees of tissue damage in the spleen, liver, kidneys, and intestines of individuals with *P. rettgeri*. Challenge experiments results confirmed that the *P. rettgeri* caused morbidity and mortality in healthy Chinese soft-shelled turtles, reproducing similar clinical symptoms of naturally infected individuals. And, its mortality rate was up to 91% in the highest concentration group. Screening of eight virulence-associated genes results revealed that this *P. rettgeri* strain carried virulence factors including invasion protein gene, alpha-hemolysin, and others which were considered to contribute to pathogenicity. Antibiotic susceptibility testing showed that the bacterium was sensitive to amikacin and ciprofloxacin, which may be effective therapeutic options. In conclusion, this bacterium *P. rettgeri* was the pathogen that caused this disease in *Pelodiscus sinensis*. These results provide valuable research basis for the disease prevention and control of *Pelodiscus sinensis* farming.

## Simple Summary

In this study, one bacterium strain *Providencia rettgeri* (*P. rettgeri*) was identified as the pathogen of diseased Chinese soft-shelled turtles (*Pelodiscus sinensis*) with the symptoms of body surface hemorrhages, claws and tail tips necrosis, and bleeding spots in visceral tissues. This bacterium led to morbidity and mortality in healthy Chinese soft-shelled turtles and showed similar clinical symptoms to individuals with spontaneous onset of the disease after challenge experiments. It contains important virulence genes such as the invasion protein, alpha-hemolysin, etc. And, this *Providencia rettgeri* was sensitive to amikacin and ciprofloxacin. The results are helpful for the research and prevention of diseases in Chinese soft-shelled turtle farming.

## 1. Introduction

Chinese soft-shelled turtle (*Pelodiscus sinensis*) is the most widely farmed reptile worldwide, particularly in China, Asia, due to its medicinal and nutritional values [1,2]. Rising market demand has rapidly driven the expansion of this industry. In 2024, the farmed production of Chinese soft-shelled turtles reached 541,589 tons and increased steadily each year [3]. However, the industry’s expansion, along with high-density and high-intensity farming systems, has exacerbated stress and increased the vulnerability of Chinese soft-shelled turtles in individual and population levels. Therefore, infectious diseases frequently break out in the breeding practice. Similarly to other aquatic species, diseases affecting Chinese soft-shelled turtles include viral [4,5,6,7,8], fungal [9], and parasitic infections [10]. Among these, diseases caused by the bacterial pathogens are the most common and have caused substantial economic losses in their farming in recent years, with impacts steadily increasing alongside the continued expansion and intensification of the industry [11]. Bacteria such as *Aeromonas hydrophila*, *Bacillus cereus*, and *Morganella morganii* cause various symptomatic diseases that result in high mortality in Chinese soft-shelled turtle populations [5,6,7,8].

*Providencia rettgeri* (*P. rettgeri*) is a Gram-negative bacteria, belonging to the genus *Providencia*, family *Enterobacteriaceae* [12]. It can adhere to exfoliated urinary cells and uroepithelial mucosal cells, thereby causing urinary tract infection [13]. In addition to causing urinary tract infections, it can cause severe diseases such as diarrhea, septicemia, sepsis, meningitis, and ocular and systemic infections under certain conditions [14,15]. Therefore, *P. rettgeri* is considered an opportunistic pathogen in humans and animals. Infections caused by this bacterium are prevalent under conditions involving immunocompromised individuals or environments with poor hygiene, where host immune defenses are weakened or presenting predisposing factors. In aquatic animals, several reports have documented the disease-causing potential of this bacterium [16,17]. Hereon, a disease associated with a mortality rate over 30% and that caused relatively large economic losses has emerged in one Chinese soft-shelled turtles farm. The diseased Chinese soft-shelled turtles exhibited body surface hemorrhages, claws and tail tips necrosis, and bleeding spots in visceral tissues. One *P. rettgeri* strain was isolated from clinical specimens of the diseased individuals. Its histopathological effects, pathogenicity, virulence-associated genes, and drug sensitivity were analyzed. These findings provide the valuable data to guide clinical antibiotic selection and to enhance the prevention and control of *P. rettgeri* in Chinese soft-shelled turtle aquaculture.

## 2. Materials and Methods

### 2.1. Experiment Animals

Twenty diseased Chinese soft-shelled turtles (300 ± 20 g) were sampled from one farm in Hubei Province. Healthy individuals (129 ± 2 g) were collected from another farm in Hubei Province. All turtles were temporary reared in a circulating water system at 28 ± 1 °C and fed with commercial food twice a day for 14 days. Turtles were randomly selected and the absence of abnormal behavior and surface injuries was tested to ensure their healthy status before the infection experiment. All the experimental procedures involving animals complied with the experimental animal guidelines of Yangtze River Fisheries Research Institute (approval number YFI2024MY01).

### 2.2. Symptom Observations and Bacterial Isolation

Diseased Chinese soft-shelled turtles exhibiting dermal symptoms were carefully examined through visual inspection. Pathological changes in diseased individuals were observed post-necropsy, with healthy turtles used as controls for comparative analysis. Liver tissue samples were collected and cultured on brain heart infusion (BHI) agar plates (HopeBio, Qingdao, China) in the incubator with normal atmosphere at 28 °C for at least 24 h. Dominant colonies were selected and subcultured on fresh BHI agar plates at 28 °C overnight to obtain pure monoclonal colonies. A single pure colony was then inoculated into liquid BHI medium and incubated at 28 °C with shaking at 200 rpm until its bacterial density reached an OD600 of approximately 0.5. The liquid bacterial culture was stored in 25% glycerol at –80 °C and designated as CSST23.

### 2.3. Bacterial Morphological Observation

The bacterial strain CSST23 was incubated on the BHI agar plates at 28 °C overnight for observation of clonal morphology. Separately, they were suspended in phosphate-buffered saline (PBS), smeared onto slides and air-dried, then performed Gram staining by a commercial kit (Jiancheng, Nanjing, China). The morphological characteristics of stained cells were examined under a light microscope (Olympus, Tokyo, Japan) of 2000× magnification with oil immersion. In addition, another part of the bacteria was carried out under scanning electron microscope (SEM, Hitachi, Tokyo, Japan) observation (35,000× magnification) after a series of processes including being fixed in 2.5% glutaraldehyde, dehydrated through the ethanol gradient, and dried.

### 2.4. Analysis of Physical and Chemical Properties

The physical and chemical properties of strain CSST23 were analyzed using the Bacterial Automatic Identification System (Biolog, Hayward, CA, USA). A single colony of CSST23 was suspended in the corresponding IF-A inoculation solution and bedded into the GEN III identification plates with 100 µL each well. Then, the GEN III plates were placed into the Biolog automatic identification system to incubate at 28 °C for 24 h, and then phenotypic analysis.

### 2.5. Analysis of 16S rRNA Sequence

The bacterial DNA of strain CSST23 was obtained by bacterial genomic DNA extracted kit (Tiangen, Beijing, China). The bacterial 16S rRNA sequence was used to identify the taxonomic classification of strain CSST23. Bacterial universal primers 27F and 1492R were shown in Table 1. The polymerase chain reaction (PCR) amplification was performed following the protocol described by Xue et al. [18]. The PCR products were sequenced by Tianyihuiyuan Biotechnology. The obtained sequence was placed in the NCBI database and compared for similarity with other sequences by the BLAST (version 2.17.0) tool (https://blast.ncbi.nlm.nih.gov/Blast.cgi accessed on 15 June 2025). A phylogenetic tree based on the Neighbor-Joining method with bootstrap value 1000 was constructed according to 16S rRNA sequences, including strain CSST23 and related sequences retrieved from NCBI using Molecular Evolutionary Genetics Analysis software (MEGA, version 7.0) [19].

### 2.6. Histopathological Analysis

Tissue samples of the intestine, spleen, kidney, and liver from three diseased Chinese soft-shell turtles were fixed in the 4% paraformaldehyde phosphate solution at 4 °C over 24 h, then dehydrated through a series-graded alcohol for histopathological examination. Samples embedded in the paraffin were sectioned to 5 μm slices by a Leica Biosystems RM2255 microtome (Leica, Wetzlar, Germany), and then were stained with hematoxylin and eosin (H&E) Staining Kit (Servicebio, Wuhan, China). Their pathological changes were examined and imaged using an Olympus optical microscope (Tokyo, Japan). Identical procedures were performed on tissues from the healthy Chinese soft-shelled turtles as control.

### 2.7. Pathogenicity

To assess pathogenicity of strain CSST23, healthy Chinese soft-shelled turtles were challenged by injection with different concentrations of bacterial suspension. The CSST23 strain bacterium suspension was prepared accordingly. Healthy Chinese soft-shelled turtle individuals were randomly divided into five treatment groups and one control group. Thirty individuals of each experimental group were injected with different bacterial suspensions at concentrations of 1 × 10^4^, 1 × 10^5^, 1 × 10^6^, 1 × 10^7^, and 1 × 10^8^ colony-forming units (CFU) per gram of body weight, respectively, from the base of abdominal shield with abdominal cavity infection, and the control group received the equivalent PBS. The morbidity and mortality were observed and recorded lasting for 10 days. Bacteria were isolated and identified again from the affected individuals according to the above method.

### 2.8. Virulence Gene Assay

Eight virulence-associated genes of molybdenum cofactor biosynthesis protein gene (*mogA*), Salmonella secreted effector L (*sseL*), Salmonella plasmid virulence gene A (*spvA*), magnesium transport C (*mgtC*), glycerol-manno-heptose gene A (*gmhA*), invasion protein gene (*invA*), outer membrane protein (*ompA*), and alpha-hemolysin (*hlyA*) were targeted to assess potential virulence of strain CSST23. The corresponding primer sequences and amplification conditions were shown in Table 1. Each 25 μL PCR contained 1 μL of each primer, 2 μL of template DNA, 12.5 μL of 2× Taq PCR Master Mix, and 8.5 μL of sterile double-distilled water. The amplification program was as follows: initial denaturation at 95 °C for 5 min; 35 cycles of denaturation at 94 °C for 1 min, annealing at the temperature listed in Table 1 for 45 s, and extension at 72 °C for 1 min; followed by a final extension at 72 °C for 10 min. Amplification products were verified by 1.5% agarose gel electrophoresis and then sequenced.

### 2.9. Drug Susceptibility Test

Ten antibiotics, including florfenicol, amikacin, ciprofloxacin, sulfamethoxazole, enrofloxacin, doxycycline, neomycin, and gentamicin, cephalothin, pediatric compound sulfamethoxazole (Hangwei, Hangzhou, China) were used to evaluate drug sensitivity of strain CSST23. These antibiotics were set on the nutrient agar plates as previously described [25]. Concentration of bacterial suspension was adjusted to a 0.5 McFarland standard. Drugs were applied to Mueller–Hinton agar plates (Difco, Detroit, MI, USA), followed by placement of antibiotic-impregnated disks and incubation at 28 °C for 24 h. Three antibiotic-impregnated disks of each antibiotic drug were performed. The diameters of inhibition zone surrounding each drug-sensitive test disk were measured. Antibiotic resistance was analyzed using CLSI M100 ED34 breakpoints [26], and labeled as resistant (R), moderately sensitive (M), and sensitive (S).

## 3. Results

### 3.1. Clinical Signs and Symptoms

**Figure 1:**
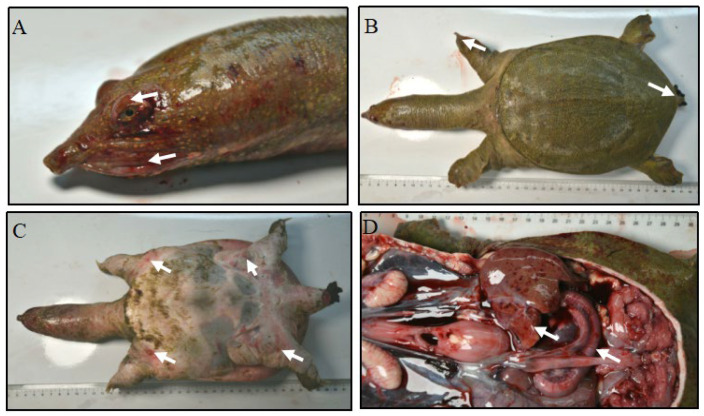
Clinical signs and symptoms observed in diseased Chinese soft-shelled turtles. (**A**) Spitting blood from the mouth and swollen eyes (white arrows). (**B**) Ulceration at the claw tips and anal prolapse (white arrows). (**C**) Edema on the ventral surface and hemorrhage on the plastron (white arrows). (**D**) Ascites in abdominal cavity, hemorrhagic spots on liver, and intestinal redness and swelling observed during autopsy (white arrows).

Diseased Chinese soft-shelled turtles exhibited oral hemorrhage and ocular swelling (Figure 1A). Ulceration was observed at the claw tips, and anal prolapse was evident (Figure 1B). Edema appeared on the ventral surface, accompanied by plastral hemorrhage (Figure 1C). Necropsy revealed ascitic fluid in the abdominal cavity, hemorrhagic lesions, and reddened, swollen, and hemorrhagic intestines (Figure 1D).

### 3.2. Morphological Observation

**Figure 2:**
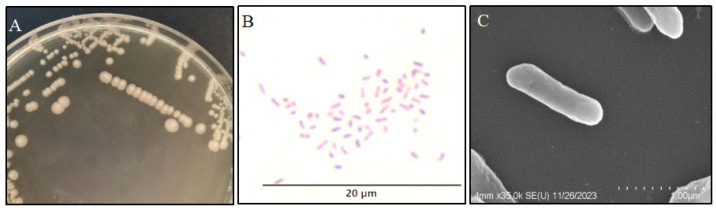
Morphological characteristics of the isolated strain CSST23. (**A**) Colony morphology. (**B**) Gram-stained image of the bacterium. (**C**) Scanning electron observation of the bacterium.

The CSST23 colonies appeared opaque and beige, with a smooth, moist surface and a circular shape featuring a slightly raised center on the BHI medium (Figure 2A). Gram staining indicated that the bacterium was Gram-negative, with a straight body and blunt ends (Figure 2B). Additionally, under SEM, individual bacterial cells appeared as elongated rods with rounded ends, lacking flagella, and measuring approximately 0.25 × 1.15 μm (Figure 2C).

### 3.3. Bacterial Biochemical Identification

Biochemical characterization of strain CSST23 was performed and the results were summarized in Table 2. According to the system database, the strain CSST23 was identified as *P. rettgeri*.

### 3.4. 16S rRNA Gene Sequence Analysis

**Figure 3:**
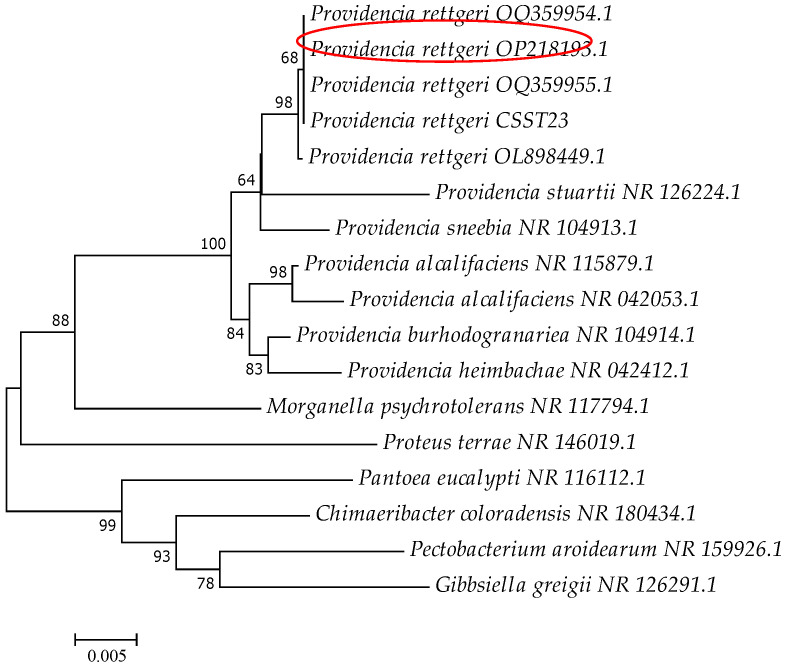
The constructed phylogenetic tree with bootstrap values based on the 16S rRNA sequences of different bacteria. Strain *Providencia rettgeri* CSST23 highlighted with a red circle.

The 16S rRNA sequence of isolated CSST23 strain was 1387 bp, corresponding to the expected size. The sequencing results were submitted to NCBI for comparison with other sequences. The results revealed that it was over 99.9% similarity with sequences such as OK090480.1, CP077260.1, and others, all of which belong to *P. rettgeri*. Sixteen sequences from different genera available in NCBI were selected to construct a Neighbor-Joining phylogenetic tree. The resulting phylogenetic tree (Figure 3) indicated that the isolated strain CSST23 clustered with members of the genus *P. rettgeri*, forming a distinct clade. Accordingly, the 16S rRNA sequence confirmed that this bacterial strain belonged to *P. rettgeri*. Combining the morphological characteristics, biochemical properties, and 16S rRNA sequencing, the isolated CSST23 was confirmed to *P. rettgeri*, a Gram-negative bacillus.

### 3.5. Histopathological Observations

**Figure 4:**
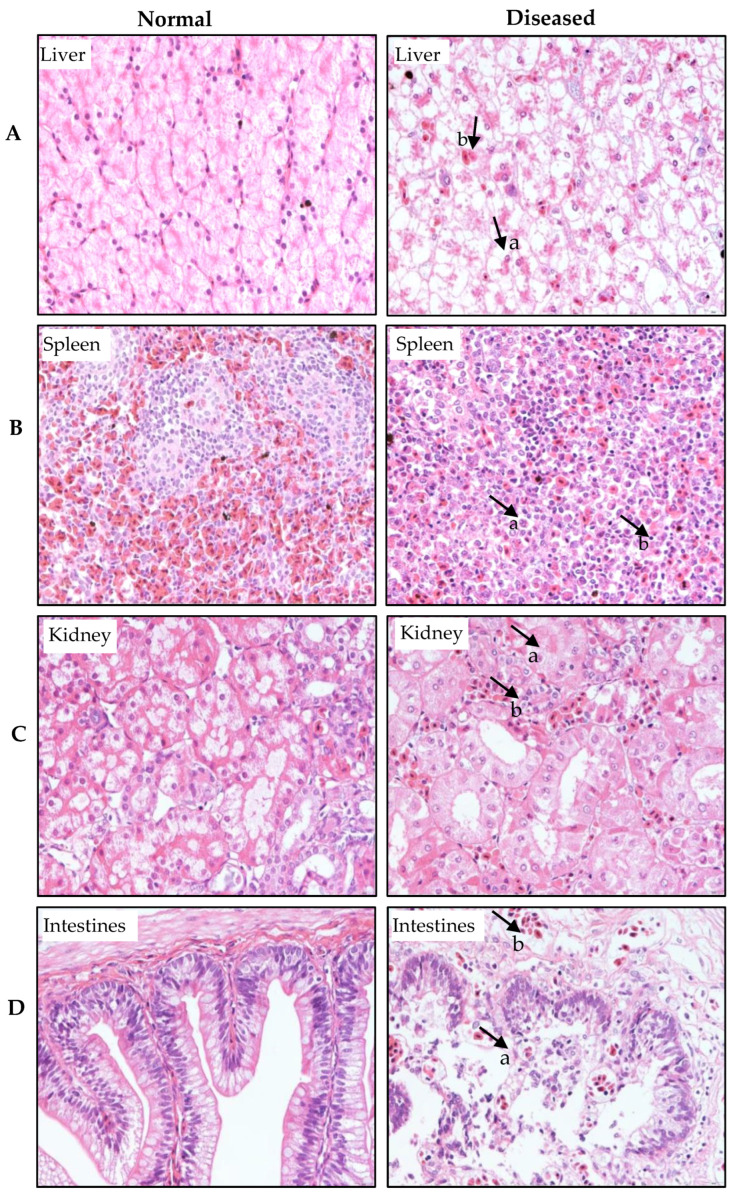
Histopathological observation of normal and diseased liver (**A**), spleen (**B**), kidney (**C**), and intestines (**D**). (**A**) Pathological changes in the liver: hepatocytes exhibited extensive swelling and necrosis (a), and hepatic sinusoids were dilated and congested, accompanied by infiltration of inflammatory cells (b). (**B**) Pathological changes in the spleen: splenic cells displayed swelling and necrosis (a), and the boundary between white and red pulp became indistinct, with infiltration of inflammatory cells (b). (**C**) Pathological changes in the kidney: renal tubular epithelial cells exhibited necrosis and exfoliation (a), alongside glomerular swelling and interstitial infiltration of lymphocytes and erythrocytes (b). (**D**) Pathological changes in the intestines included severe necrosis and exfoliation of columnar cells and goblet cells in the villous epithelium, leading to significant villous damage (a). The blood vessel layer was dilated and congested, and muscle cells exhibited necrosis and a disorganized arrangement (b). Magnification: 40×.

Histopathological examination was performed on the liver, spleen, kidney, and intestinal tissues of healthy and diseased soft-shelled turtles, with the results presented in Figure 4. In the healthy liver tissue, hepatocyte nuclei and cytoplasm were uniformly stained, exhibiting well-defined cellular structures. In contrast, the diseased liver samples revealed the extensive hepatocellular swelling and necrosis, nuclear material marginalization, and disintegration of cellular architecture (Figure 4A-a). Hepatic sinusoids were dilated and congested, with prominent infiltration of inflammatory cells into the hepatic parenchyma (Figure 4A-b). In the normal spleen, cells were evenly stained and exhibited a compact structure with clearly organized white and red pulp. However, diseased spleen tissues exhibited cellular swelling and necrosis, often resulting in complete structural loss (Figure 4B-a). The boundary between white and red pulp became indistinct, accompanied by infiltration of inflammatory cells (Figure 4B-b). In normal kidney tissue, the glomerular structure was well-defined, renal tubular epithelial cells demonstrated uniform staining, and overall architecture remained intact. In contrast, affected renal tissues exhibited tubular epithelial cell necrosis and exfoliation (Figure 4C-a), along with glomerular swelling and interstitial infiltration of lymphocytes and erythrocytes (Figure 4C-b). In healthy intestinal tissue, the villi maintained an intact structure with an orderly arrangement. In infected specimens, the columnar and goblet cells of the villous epithelium underwent severe necrosis and exfoliation, leading to significant damage to the intestinal villi (Figure 4D-a). Blood vessels in the muscular layer were dilated and congested, while muscle cells exhibited necrosis and disorganization (Figure 4D-b).

### 3.6. Pathogenicity Test

**Figure 5:**
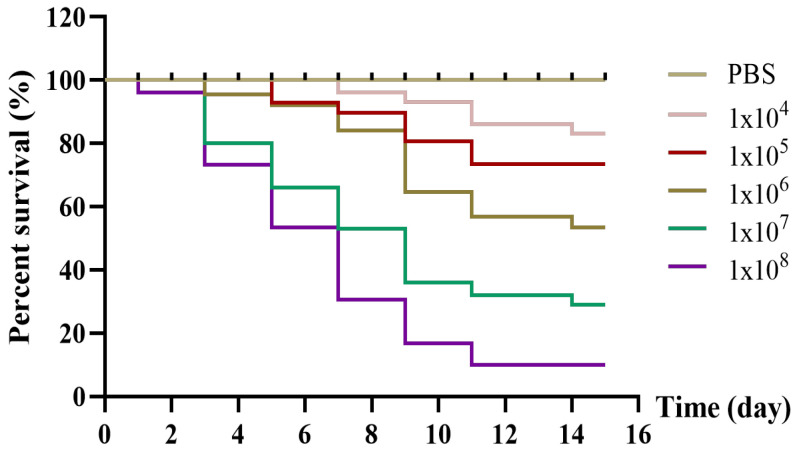
Survival rates of Chinese soft-shelled turtle challenged with different concentrations of strain CSST23 over post-infection period.

Five bacterial concentrations were used to evaluate the pathogenicity of strain CSST23 in Chinese soft-shelled turtles. As illustrated in Figure 5, mortality occurred in all experimental groups at varying rates within 14 days, while the control group had no deaths. Highest mortality rate of 91% was observed within 14 days in the group injected with 1.0 × 10^8^ CFU/g. A positive relationship was observed between bacterial concentration and mortality rate. In different infection groups, the affected individuals exhibited exactly the same symptoms as those resulting from natural illness, or some of these symptoms. Furthermore, the bacterium isolated again from the experimental infected groups was identified as *P. rettgeri* too.

### 3.7. Virulence Gene Assay

**Figure 6:**
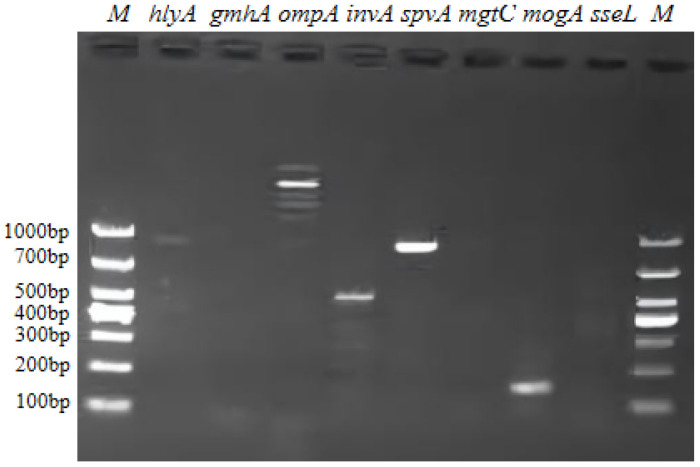
Eight virulence genes detection of *P. rettgeri* by agarose gel electrophoresis. M: DL1000 marker.

Eight virulence factor genes were screened, including *hlyA* (789 bp), *gmhA* (1000 bp), *ompA* (919 bp), *invA* (214 bp), *spvA* (432 bp), *mgtC* (200 bp), *mogA* (419 bp), and *sseL* (304 bp). The results (shown in Figure 6) showed that five genes of *mogA*, *spvA*, *invA*, *hlyA*, and *ompA* were positive, while *gmhA*, *mgtC*, and *sseL* were negative. These findings suggest that the isolated strain *P. rettgeri* CSST23 may express virulence factors encoded by *mogA*, *spvA*, *invA*, *hlyA*, and *ompA*.

### 3.8. Drug Sensitivity Analysis

The results of the antibiotic susceptibility analysis are presented in Table 3. The isolated strain *P. rettgeri* CSST23 exhibited high sensitivity to amikacin and ciprofloxacin. Resistance was observed against sulfamethoxazole, doxycycline, florfenicol, neomycin, gentamicin, enrofloxacin, cephalothin, and paediatric compound sulfamethoxazole tablets.

## 4. Discussion

Bacterial diseases pose a significant challenge in freshwater aquaculture, often resulting in high mortality rates and substantial economic losses. Both field observations and published data indicate that bacterial infections are the most prevalent health issue in Chinese soft-shelled turtle farming. Hereon, bacterial strain CSST23, identified as *P. rettgeri*, was isolated and confirmed as the pathogenic agent responsible for the disease outbreak in Chinese soft-shelled turtles.

Coupled with the development of the Chinese soft-shelled turtle farming industry raising, alteration of the aquatic farming environment often leads to an increase in the outbreak of infectious diseases, and primarily of bacterial and viral origin [7,27]. *P. rettgeri* is a Gram-negative bacterium belonging to the genus *Providencia* within the family *Enterobacteriaceae*, and usually exists in diverse environments and hosts [28,29]. The first identified occurred in Japan in 2002, it spread globally by 2015 [13], and was associated with disease outbreaks in humans and animals [30,31]. Due to its opportunistic pathogenicity and extensive multidrug resistance, *P. rettgeri* has become an increasing concern as an opportunistic pathogen in environmental contexts [13]. Additionally, *P. rettgeri* has been reported to infect multiple aquatic species, causing diseases with varying clinical symptoms. Documented hosts include *Hypophthalmichthys molitrix* (silver carp) [32], *Penaeus Chinensis* (Chinese white shrimp) [33], *Oreochromis niloticus* (Nile tilapia) [34], *Litopenaeus vannamei* (Pacific whiteleg shrimp) [35,36], *Marsupenaeus japonicus* (kuruma shrimp) [17], *Cuora flavomarginata* (yellow-margined box turtle) [12], *Trachemys scriptaelegans* (red-ear turtle) [37], *Trachemys scripta* (slider turtle) [38], *Trionyx Sinensis* (soft-shelled turtle) [39], *Ptyas mucosus* (mucosal rat snake) [40], *Varanus salvadorii* (crocodile monitor lizard) [41], *Crocodylus porosus* (Chinese alligator) [42], and juvenile farmed *Alligator mississippiensis* [43]. In this study, *P. rettgeri* was isolated from diseased farmed Chinese soft-shelled turtles. These findings suggest that the bacterium can cause widespread infections across aquatic animals and may exhibit a higher propensity to infect amphibians and reptiles compared to fish.

*P. rettgeri* causes diseases exhibiting diverse clinical symptoms in animals, including septicemia, ulcers on the abdomen, pectoral fins and head region, orbital edema, hemorrhage, and focal necrosis of the sclera. In this present study, *P. rettgeri* infection in Chinese soft-shelled turtles resulted in symptoms such as edema, ulceration, anal prolapse, and intestinal septicemia. In *Trachemys scriptaelegans*, *P. rettgeri* was associated with ophthalmia characterized by eyelid inflammation and congestion [37]. In *Cuora flavomarginata*, *P. rettgeri* infection caused no visible external symptoms; however, the liver appeared enlarged and was covered with blood vessels [12]. Additionally, in *Trachemys scripta*, *P. rettgeri* infection led to the hemorrhagic septicemia [38]. In another report, *P. rettgeri* was found to cause the visceral abscesses in Chinese soft-shelled turtles [39]. These observations indicate that symptoms induced by *P. rettgeri* vary among host species, even among those with similar taxonomic classifications. This finding further supports the complexity and diversity of bacterial diseases in aquatic animals. One possible explanation for this variation is the presence of different virulence factors carried by individual isolates.

Bacterial pathogens can produce and secrete some proteins, referred to as virulence factors. These proteins are encoded by specific genes located on bacterial chromosomes or mobile genetic elements. Understanding virulence factors is essential for elucidating bacterial pathogenesis, developing therapeutics, and designing vaccines [44]. The pathogenicity of bacteria in susceptible hosts is influenced by multiple virulence factors that act independently or synergistically at various stages of infection. Based on their mechanisms and functions, virulence factors are commonly classified into five categories: membrane proteins (involved in adhesion, invasion, colonization, and surface outer membrane structure), secretory proteins (including immune response inhibitors, toxins, and toxin transporters), capsule, cell wall and outer membrane components, and other factors, such as biofilm, iron acquisition, and the PhoP/PhoQ two-component system. These factors play distinct roles and contribute to varied infection symptoms during the disease process [45]. *P. rettgeri* exhibits several virulence characteristics, including adherence, invasion, motility, protein toxin production, diarrhea induction, and dissemination within the host, all of which contribute to the manifestation of various symptoms [46]. This study detected five virulence factor genes of bacteria, *spvA*, *mogA*, *invA*, *hlyA*, and *ompA* in the CSST23 strain of *P. rettgeri*. The *invA* gene facilitates invading the intestinal epithelial cells of host [47]. The *hlyA* gene encodes a secreted protein toxin that disrupts host cell signal transduction and cytokine production, damages tissues, penetrates mucosal barriers, facilitates the release of nutrients from host cells, and impairs immune cell functions [48,49]. The *ompA* gene contributes to adhesion and invasion [50]. The gene *spvA*, a member of the *spv* gene family, regulates host cell death [51]. Collectively, these virulence factors are involved in adhesion, invasion, intracellular survival, toxin production, and immune response inhibition and are primarily associated with the pathogenesis of Gram-negative bacteria. The virulence genes of *gmhA*, *mgtC*, and *sseL* were not detected in this *P. rettgeri* stain. We speculated that, on one hand, they might not exist in this bacterium stain and, on the other hand, it could be that the PCR primers are insufficient. Further in-depth research on these virulence genes is still needed in the future.

*P. rettgeri*, a significant conditional and opportunistic pathogen, causes infections under specific environmental or host-related conditions. It has been implicated in high mortality rates among intensively farmed *Alligator mississippiensis* at commercial aquaculture facilities [43]. Currently, Chinese soft-shelled turtles are initially reared in greenhouses using recirculating aquaculture systems during their early growth stage and are subsequently transferred to outdoor ponds until they reach the marketable size. This farming model is widely adopted in China; however, it contributes to water pollution and compromises the immune defense of Chinese soft-shelled turtles, leading to a high occurrence of sudden infectious diseases [15,52]. Fan et al. [53] reported that at a specific breeding facility, Chinese soft-shelled turtles reared under natural conditions were susceptible to bacterial infections, primarily from June to October. In contrast, those raised in greenhouses were vulnerable year-round. Additionally, Liu et al. [54] observed distinct differences in the intestinal microbiota of Chinese soft-shelled turtles between greenhouse and pond culture environments. In this study, diseased Chinese soft-shelled turtles were reared in a factory-based breeding system characterized by commercial feed usage and high stocking density. These conditions may elevate the risk of bacterial infection in farmed individuals. Consequently, under such conditions, the conditional/opportunistic pathogen *P. rettgeri*, which is prevalent in water and the surrounding environment, has the potential to cause infection in Chinese soft-shelled turtles. Although such infections are infrequent, the resulting losses are substantial and present significant challenges to the sustainability and expansion of the Chinese soft-shelled turtle farming industry. The limitation of this study was that it only analyzed one incident of this *P. rettgeri* bacterium infection in a certain farm. In the future, more attention should be paid to the prevalence of this bacterium among Chinese soft-shelled turtles.

Antibiotic and vaccines remain the primary strategies for treatment and prevention of bacterial infections in aquatic animals. Currently, the management of *P. rettgeri* predominately relies on the chemical antibiotic [24]. Review of previous studies indicates that the *P. rettgeri* from various aquatic animals are generally sensitive to florfenicol, enrofloxacin, and imipenem [24,35,36,37,39]. In this study, drug susceptibility testing showed this *P. rettgeri* was sensitive to amikacin and ciprofloxacin but resistant to sulfamethoxazole, doxycycline, florfenicol, neomycin, gentamicin, enrofloxacin, cephalothin, and pediatric compound sulfamethoxazole. Antibiotic resistance testing showed that this *P. rettgeri* exhibited a broad spectrum of antibiotics resistance. This finding may be attributed not only to the increasing antibiotic resistance in *P. rettgeri* [55] but also to the presence of diverse antibiotic resistance genes in various isolates [56]. Antibiotic misuse and residual drug accumulation have become critical concerns for environmental and food safety. Notably, sanguinarine (SAG), a compound derived from traditional Chinese herbal plants, has recently demonstrated antibacterial activity against *P. rettgeri* in vitro. Its result revealed that SAG exhibited strong inhibitory activity against *P. rettgeri*, primarily by disrupting cell membrane integrity and inactivating biofilm-associated cells [57]. Additionally, a novel multi-epitope recombinant vaccine targeting *P. rettgeri* was developed using an immunoinformatics-based approach [58]. These advances suggest that more effective preventive strategies against this pathogen may become available in the future.

## 5. Conclusions

This study confirmed that one pathogenic strain of *P. rettgeri* was responsible for the disease outbreak in farmed Chinese soft-shelled turtles. Amikacin and ciprofloxacin may serve as effective therapeutic options. To support the development of Chinese soft-shelled turtle aquaculture, it is essential to strengthen daily farm management, improve rearing conditions, reduce susceptibility factors associated with environmental stress, and ensure the safe and rational use of antibiotic or the adoption of novel approaches to prevent and control *P. rettgeri* infections.

## Figures and Tables

**Table 1:** Primer sequences used for PCR amplification in this study.

Genes	Primer Sequence (5′–3′)	Size (bp)	Tm (°C)	Sources
16S rRNA	27F: AGAGTTTGATCCTGGCTCAG	1465	56	[18]
	1492R: TACGGCTACCTTGTTACGACT T			
*mogA*	F: ATTGGCTTAGTTTCTATCTCCG	419	52	[20]
	R: CCTTCCAGCGTTTCTTTGA			
*sseL*	F: CTATCCTATTGGGCTTAT	304	50	
	R: GTTGGGTACATTGTTCTG			
*mgtC*	F: CGACGATCATTATTCTTTGC	200	53	
	R: GACCGAACCTAACCCTTGT			
*spvA*	F: GCTAACTGTCGGGCAAAG	432	53	
	R: GGACAATGGCACGAACCT			
*invA*	F: TTGTCACCGTGGTCCAGTTTATCG	214	57.2	[21]
	R: TTCATCGCACCGTCAAAGGAACC			
*gmhA*	F: CGGAATTCTGATGCTAGGGATAACTTG	1000	60	[22]
	R: CCGAGCTCCGGGGAAGCAATGGTTAAG			
*hlyA*	F: CGTGGACACAGCTGCCAGCA	789	54	[23]
	R: TGCAGCGTGGCGGGCATCAT			
*ompA*	F: AGCTATCGCGATTGCAGTG	919	58	[24]
	R: GGTGTTGCCAGTTAACCGG			

**Table 2:** Biochemical properties of CSST23 strain.

Reaction Item	Result *	Reaction Item	Result *
Negative control	N	Gelatin	N
Dextrin	B	Glycyl-L-Proline	B
D-Maltose	N	L-Alanine	B
D-Trehalose	N	L-Arginine	N
D-Cellobiose	N	L-Aspartic Acid	B
Gentiobiose	N	L-Glutamic Acid	B
Sucrose	N	L-Histidine	B
D-Turanose	N	L-Pyroglutamic Acid	B
Stachyose	N	L-Serine	B
Positive control	P	Lincomycin	B
Acidic pH 6	P	Guanidine HCl	P
Acidic pH 5	B	Niaproof 4	P
D-Raffinose	N	Pectin	N
α-D-Lactose	N	D-Galacturonic Acid	N
D-Melibiose	N	L-Galactoric Acid Lactone	N
β-Methyl-D-Glucoside	B	D-GalactoricAcid	B
D-Salicin	B	D-Glucuroric Acid	N
N-Acetyl-D-Glucosamine	B	Glucuronamide	N
N-Acetyl-β-Mannosamine	B	Mucic Acid	N
N-Acetyl-D-Galactosamine	B	Quinic Acid	N
N-Acetylneurarminic Acid	B	D-Saccharic Acid	N
1% NaCl	P	Vancomycin	P
4% NaCl	N	Tetrazolium Violet	P
8% NaCl	N	Tetrazolium Blue	P
α-D-Glucose	B	P-Hydroxyphenylacetic Acid	B
D-Mannose	B	Methyl Pyruvate	N
D-Fructose	B	D-Lactic Acid Methyl Ester	N
D-Galactose	P	Lactic Acid	N
3-Methyl Glucose	N	Citric Acid	B
D-Fucose	N	α-Ketoglutaric Acid	B
L-Fucose	N	D-Malic Acid	B
L-Rhamnose	B	L-Malic Acid	B
Inosine	B	Bromosuccinic Acid	B
1% Sodium Lactate	B	Nalidixic Acid	B
Fusidic Acid	B	Lithium Chloride	B
D-Serine	P	Potassium Tellurite	N
D-Sorbitol	N	Tween 40	N
D-Mannitol	B	Y-Aminobutyric Acid	N
D-Arabitol	B	α-Hydroxybutyric Acid	N
Myo-lnosito	B	β-Hydroxy-D, L-butyric Acid	N
Glycerol	B	α-Ketobutyric Acid	N
D-Glucose-6-PO_4_	B	Acetoacetic Acid	B
D-Frutose-6-PO_4_	P	Propionic Acid	N
D-Aspartic Acid	B	Acetic Acid	B
D-Serine	N	Formic Acid	N
Troleandomycin	B	Aztreonam	P
Rifamycin SV	P	Sodium Butyrate	B
Minocycline	P	Sodium Bromate	N

Notes: * means Abbreviations: P means positive; N means negative; B means borderline.

**Table 3:** Results of the drug sensitivity assay.

Drug	Content (μg/Disk)	Inhibition Zone (mm)	Sensitivity
Amikacin	10	17	S
Ciprofloxacin	10	16	S
Sulfamethoxazole	10	0	R
Doxycycline	10	0	R
Florfenicol	30	0	R
Neomycin	10	10	R
Gentamicin	10	0	R
Enrofloxacin	10	10	R
Cephalothin	30	0	R
Paediatric Compound Sulfamethoxazole	10	0	R

## Data Availability

The original contributions presented in this study are included in the article. Further inquiries can be directed to the corresponding author.
